# The inguinal hernia management in Costa Rica according to a survey between surgeons: result comparison with 2018 International Groin Hernia Guidelines

**DOI:** 10.1186/s12893-021-01156-9

**Published:** 2021-03-21

**Authors:** Jesús Martínez-Hoed, Katherine Cordero-Bermúdez, Providencia García-Pastor, Salvador Pous-Serrano, José A. Ortiz-Cubero

**Affiliations:** 1 General Surgery Department, R. A. Calderón Guardia Hospital, San José, Costa Rica; 2 Integrated Group for Complex Hernia Treatment, Calderón Guardia Hospital, San José, Costa Rica; 3 General Surgery, Metropolitan Hospital, San José, Costa Rica; 4 Costa Rica Hernia Association, San José, Costa Rica; 5 General Surgery, Clínica Bíblica Hospital, San José, Costa Rica; 6 General Surgery At Hospital Politécnico y Universitario La Fe, Valencia, Spain; 7 Short Stay and Wall Surgery Unit, Hospital Politécnico y Universitario La Fe, Valencia, Spain

**Keywords:** Groin hernia, Inguinal hernia, Inguinal hernioplasty, Survey, Clinical guideline

## Abstract

**Background:**

Inguinal hernia surgery is a frequent procedure among general surgeons in Costa Rica, but the management and technique are not uniform. The International Guideline for Groin Hernia management was published in 2018 to standardize the inguinal hernia surgery, but the diffusion of the guidelines and its adherence have been extremely varied.

**Purpose:**

Collect and analyze the current reality regarding groin hernia management in Costa Rica. Secondly evaluate the diffusion and development comparing it to the guideline’s recommendations.

**Method:**

Questionnaire of 42 single and multiple answer questions according to the topics of the International Guideline directed to general surgeons. Diffusion of the inquiry through surgical and hernia association chats and email. Timeframe June–December 2019.

**Results:**

64 surveys were collected, which is a representative number of the general surgeons national college. The most frequent procedure between these was the abdominal wall surgery. Every surgeon did more than 52 groin hernia surgeries in one year, most of them outpatients. The epidural anesthesia was used the most and Lichtenstein’s technique was the most frequently used (64%). 68% of the surgeons know how to perform a minimally invasive inguinal hernia surgery but with variable volumes. 38% of participants considered themselves experts in groin hernia management and 52% did not know the 2018 International Guideline. The recommendations of such guideline are followed only partially.

**Conclusions:**

The 2018 Hernia Surge International Guidelines have low diffusion among Costa Rican surgeons. The laparoscopic approach is widely accepted but there are no studies to assess the results and the quality. There should be protocols and studies adapted to Costa Rica’s national situation.

**Supplementary Information:**

The online version contains supplementary material available at 10.1186/s12893-021-01156-9.

## Background

Worldwide, more than 20 million inguinal hernias are operated each year, [1] in Costa Rica (CR) this surgery is a quite frequent procedure for the general surgeon. Our country has 5 million habitants and possesses a strong public health and social security system. The exact number of inguinal hernia repair is unknown but most of them are performed within the public health system. In recent years the interest in abdominal wall hernias has increased, as is reflected in the number of international publications and consensus guidelines about hernia management and particularly in inguinal hernia (IH) [2]. Costa Rican surgeons have also shown the same interest.

The general surgeon daily confronts the challenge of the IH but there are a lot of surgery techniques available with great variation in outcomes, which are also influenced by the type of hernia and the surgeon’s expertise [3, 4]. Even more, there is not a perfect surgical approach for all the inguinal hernias, the laparoscopic approach has not been standardized and the general management of IH is not uniform among surgeons even in the same country [5].

In 2018, the first International Groin Management Guideline was published by the Hernia Surge Group. The guide contains 87 recommendations submitted by international consensus and investigation. Its authors were 54 experts from 5 continental hernia societies and 2 endoscopic surgery associations. The Latin America representation was the American Hernia Society, and none of the authors were a practicing surgeon in Latin American [1].

To achieve generalization of a guideline the best scientific evidence must be used, the guideline must represent reality and a wide diffusion is necessary [6, 7]. Despite being unique in its forward-oriented approach, the high scientific value, and including the opinion of worldwide experts, the International Groin Management guide is not widely known [1, 2]. It is unknown if surgeons in CR are aware of the international guidelines or if they apply it within their daily practice. Its expected that a clinical guideline like this will standardize treatment and improve general results.

Foremost, this study was based on a survey of opinions directed to Costa Rican general surgeons concerning the topics of the 2018 guidelines. The main objective was to know and analyze the reality of the groin hernia management in CR. Secondly, compare them to the guideline's recommendations.

## Methods

An opinion poll was created by the authors and directed to general surgeons and residents. The questions were reviewed by external general surgeons not related to the investigation. The questionnaire format was similar to the Spanish national survey of the IH management which was accepted and is waiting publication [8].

The 42 single and multiple answer questions were based on the guideline’s recommendations trying to represent the national management (Additional file 1). The survey was distributed between August and December 2019 using Google Form® online platform. The diffusion was made through the CR Hernia Association and the CR Surgery Association, electronic media and email.

The information was gathered using Microsoft® Excel MAC version 16.16.19 with dichotomous splitting of multiple answers. A descriptive analysis was performed with IBM® SPSS Statistics® version 26.0 using frequencies and percentages for qualitative variables and association with Chi square and Fisher exact test among those questions that methodologically allowed the relation. The related questions were: 3 with 6, 13, 22, 37 and 41. Question 37 with 4, 6, 10, 13, 14, 21, 22, 25, 27 and 39. Question 41 with 4, 5, 6, 7, 9, 10, 13, 14, 15, 16, 17, 18, 21, 22, 25, 26, 27, 30, 31, 36, 37, 39 and 40.

## Survey results

**Fig. 1:**
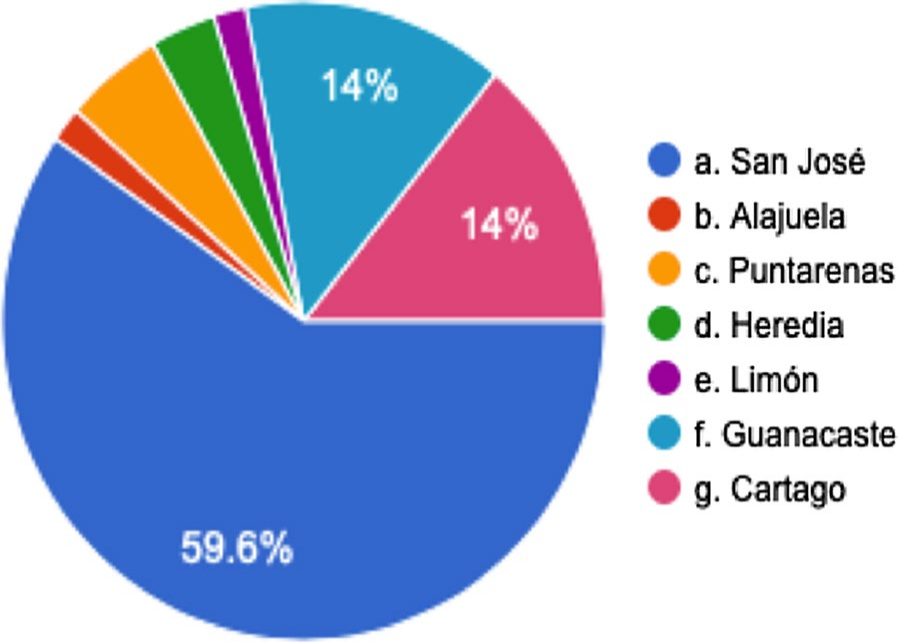
Survey answer distribution by Costa Rican provinces

The surveys obtained 64 polls. 7 of them were incomplete, but the results were calculated taking this into account. 61 surveys were completed by attending surgeons and 3 by residents. This last group was excluded from the analysis because they were not representative. The distribution of responses by states is found in Fig. 1. In the Costa Rican College of Physicians and Surgeons there are 351 general surgeons subscribed. Approximately half of them belong to general surgery subspecialties. The exact number of surgeons that perform IH repair is unknown. There are 100 active surgeons in the Costa Rican Hernia Association and 118 in the CR Surgery Association, the majority subscribed in both associations. These associations were the principal diffusion points for the survey. We tried to distribute the survey among the most active surgeons for the IH management and received more than 50% participation, which is considered representative.

## Characteristics of the surgical practice

**Fig. 2:**
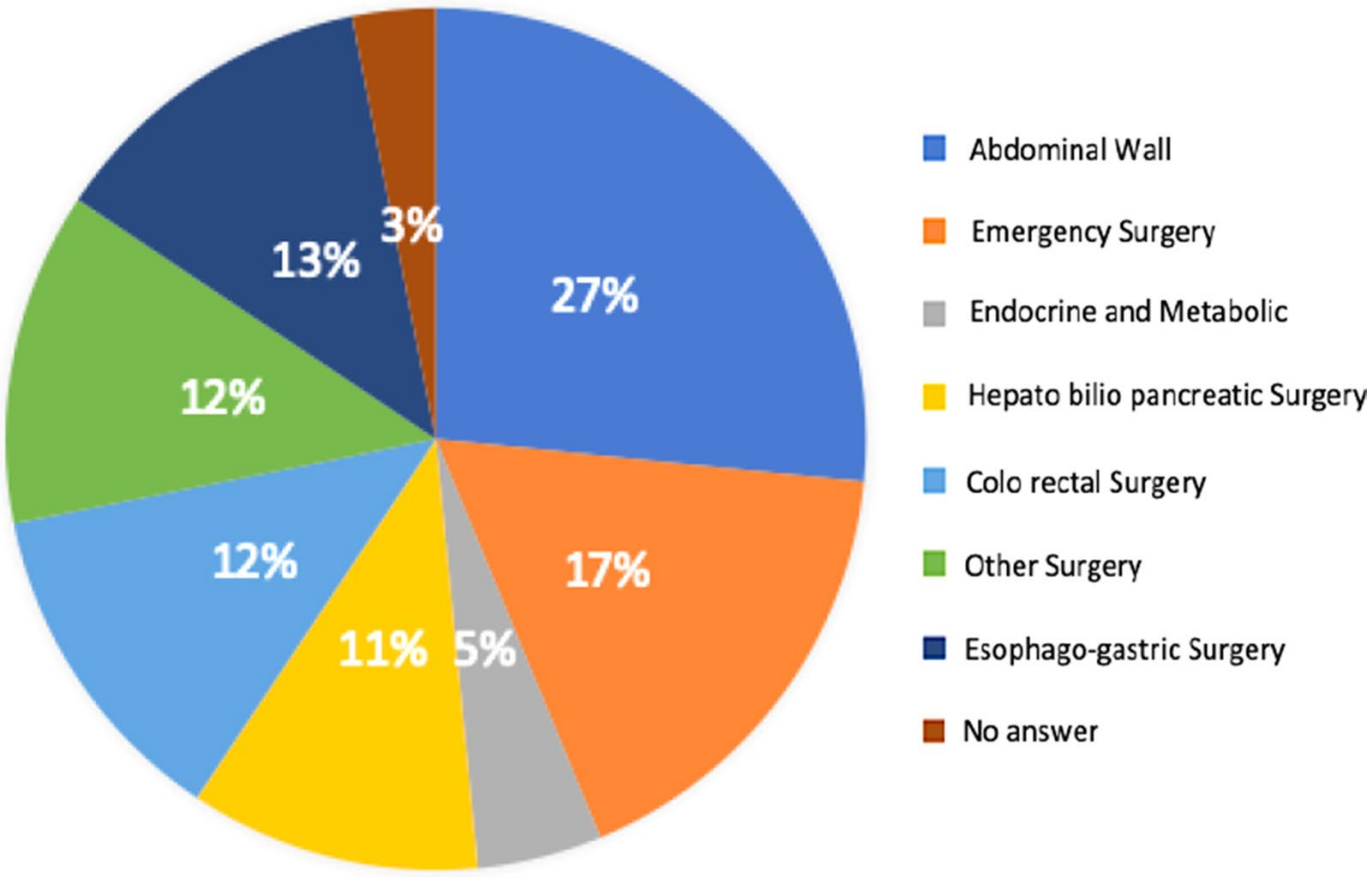
Distribution of the most common surgery performed by each surgeon

**Fig. 3:**
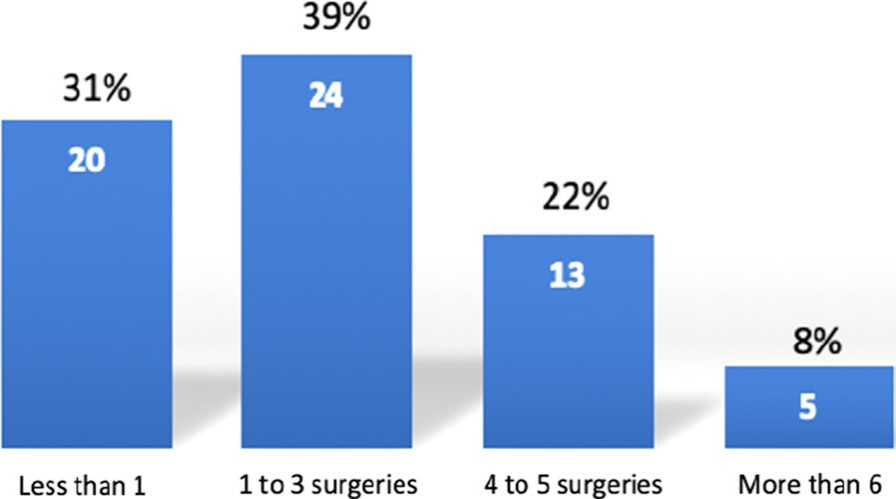
Distribution of inguinal hernia surgery volume performed weekly for each participant

Costa Rican surgeons work privately, in public health or both. Our results showed that 17 (28%) operate exclusively in public hospitals, 12% in private hospitals and 60% in both, public and private hospitals. The experience as specialists ranged from “more than 11 years” in 51% (31 surgeons), 31% between “5 to 10 years of experience”, and 18% “less than 5 years of experience”. The most common surgery performed by each surgeon is found in Fig. 2 and the volume of inguinal hernia surgery performed weekly for each participant are shown in Fig. 3.

## Preoperative management of patient with inguinal hernia

The most used IH classification was “Direct or Indirect” (66%), followed by Nyhus with 15%, EHS at 13%, “no classification” 6% and “Gilbert and Others classification” with 0%. The watch and wait management in a young male patient with a small asymptomatic or minimally symptomatic inguinal hernia, with observation and periodic appointments, was agreed by only 35% of those surveyed. 88% of participants did not feel comfortable ordering surgery on a young male patient with an asymptomatic and no palpable hernia with a diagnosis made by ultrasound. For women patients with IH, “observation” without surgery was ruled out by 90% of the surgeons.

## Exclusive dedication to inguinal hernia repair and anesthetic management

16% of the participants worked in hospitals with a team dedicated to hernias, but the majority 48 (79%) indicated that in their hospitals all surgeons performed IH surgery, and 5% indicated that only residents treat IH. The surgery was performed mostly in ambulatory mode 96% (59 participants), with spinal anesthesia in 76% (45 participants). 26% performed IH surgery with general anesthesia and 0% local anesthesia and sedation. The use of prophylactic antibiotics was indicated by 56%, for a clean and elective surgery.

When asked about local anesthesia and sedation, 69% (42 participants) did not normally use it, 19% used it only in elderly patients with important co-morbidities, and 11% used it in young patients with small hernias. 67% of the participants knew how to perform an open inguinal hernioplasty with local anesthesia.

The nerve block during IH surgery was performed by the anesthesiologist in 29% of cases and 30% by the surgeon, but the majority (41%) only infiltrated skin and subcutaneous tissue. With general anesthesia 68% (41 participants) reinforced the post-operational analgesic with local anesthetics.

## Surgical indications, techniques and volume for inguinal hernia repair

The most common surgical technique for inguinal hernias was Lichenstein (64%), followed by TAPP or TEP (26%) and the Rutkow Robbins (mesh-cone) technique represented a 10%. The “Nyhus techniques and other open preperitoneal” and “Shouldice and other anatomical techniques” were not selected.

In conventional open surgery, 53% never used a mesh cone, 26% sometimes and 21% continually. The resection of the hernia sac was accomplished “only if it is large” by 45% of participants, 21% never resect it, and 34% always do. The 58% (35 participants) do the mesh fixation to the pubic tubercle periosteum yet. The different mesh fixations in open surgery are described in Table 1. The Progrip® mesh was used by 58% of the participants.

**Table 1:** The mesh fixations preference in open surgery

Mesh fixation	Percentage (%)
Polyamide suture (Nylon®)	29
Long absorption suture (example PDX®)	26
Polypropylene	20
Auto adhesive mesh (Progrip ®)	16
Short absorption suture (example Vicryl®)	10
Cyanoacrylate	0
No mesh fixation	0

In women with elective femoral hernias, the preferred repair was the Lichtenstein with femoral modification in 42%, TAPP or TEP in 35%, McVay repair with 13%, and mesh cone in the femoral orifice in 11%. The posterior open approaches were not represented (0%). 95% of the participants performed the same surgical approach for both men and women.

68% of surveyed surgeons do inguinal hernia minimally invasive surgery (IHMIS) and the number of cases per week were as follows: 21 surgeons (36%) had less than 1 case a week, 10 surgeons (17%) performed one IHMIS per week, 7 (12%) operated between 2 and 3 cases per week, 2 surgeons (3%) intervened in more than 3 cases per week. 32% of the participants (19 surgeons) indicated they did not perform IHMIS and 2 surveys did not answer this question.

At the participating surgeon’s hospitals, 8% (5 surveys) did not perform IHMIS and 62% (37 participants) performed very few cases. The remaining 18% indicated that less than half of cases were done laparoscopically, but 7 surgeons (12%) responded that more than half of IH cases in their hospitals were done using IHMIS. The surgery indications for IHMIS were as follows: for bilateral IH, 86% agreed with this indication, in recurred IH 72%, in IH in athlete patients 52%, in women with IH 41% and in primary unilateral IH in men 41% agreed. There was a large lack of experience for open pre-peritoneal hernioplasty at 71% although 15% used it in cases of recurrent previous anterior hernioplasty, 9% (6 surgeons) always used it as a standard and 5% used only in emergencies.

## Postoperative chronic pain and emergency repair

Regarding chronic pain, the majority (52 participants or 86%) responded “Yes, there are cases, but not many, although there are no incidence studies”. The remaining 14% indicated that “there are no cases of chronic pain, although we do not have incidence studies.” The participants chose neither “We have studies and it is less than 10%” nor “We have studies and it is less than 15%.” For incarcerated IH, 84% usually perform conventional open surgery, 11% use a laparoscopic approach and 5% prefer the open pre peritoneal approach. If the hernia is strangulated, 66% “depending on the contamination use a mesh”, 20% “Always use a mesh” and 9% “Avoided using a mesh because of the risk of infection.” On this question, none of the participants chose the option “I don’t operate emergency cases” but there were 3 surveys in which this question was not answered.

## Continuous education and use of the international guideline

Regarding knowledge of the 2018 International Guideline, 52% indicated they “had not heard of it”, 28% “had read some of it” and 18% “had read it and used it as a reference.” None of the participants chose the “I have read the guide and I don’t like it”. Only 1 survey (2%) did not answer the question.

With regards to training and keeping up to date with IH surgery, 64% had taken courses, and 29% would like to be more updated. While 2% indicated they did not need to learn more, another 2% “were not interested in this pathology.” This question was left blank on 2 surveys or 3%. Even so, 92% responded they wished to improve their IH surgical technique, and 92% also opined that all surgeons who performed inguinal hernia surgery, should know how to resolve it laparoscopically.

**Fig. 4:**
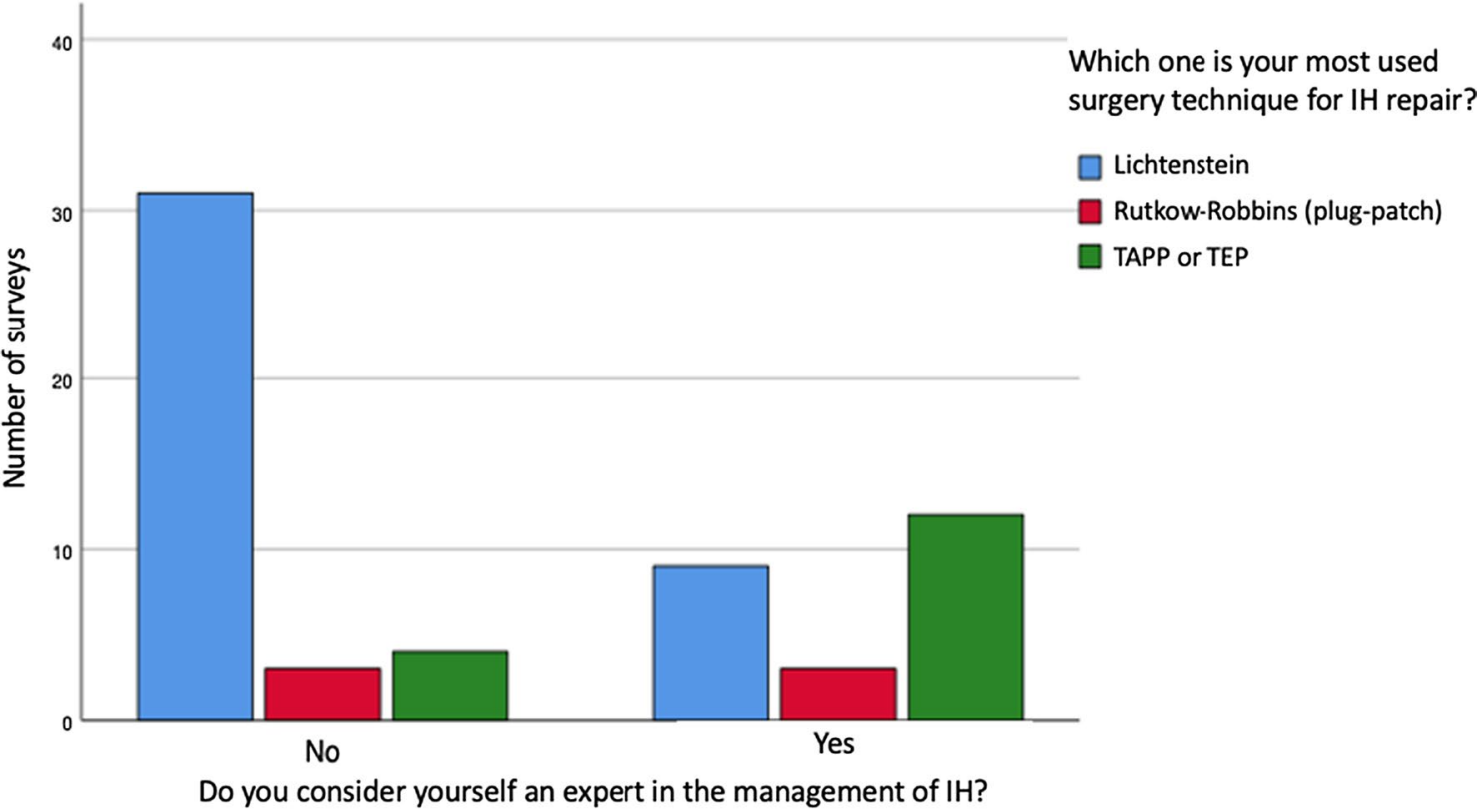
Association between “to consider themselves as an expert in the management of IH” and “the most used surgery technique for IH repair”

Among the surveyed surgeons, 17% consider themselves as expert in abdominal wall hernias management, and 38% as expert in IH treatment. A statistically significant association was found (p < 0.05) between considering themselves as expert in the management of hernia inguinal and considering themselves as expert in abdominal wall hernia management. (c^2^(fd 1) = 21.2, p < 0.05, Phi = 0.58, V Cramer = 0.58). Likewise, in considering oneself an expert in the treatment of IH and the most used surgery technique for IH repair. (c^2^(fd 2) = 13.6, p < 0.05, Phi = 0.47, V Cramer = 0.47). Figure 4 shows the frequency of each category.

**Fig. 5:**
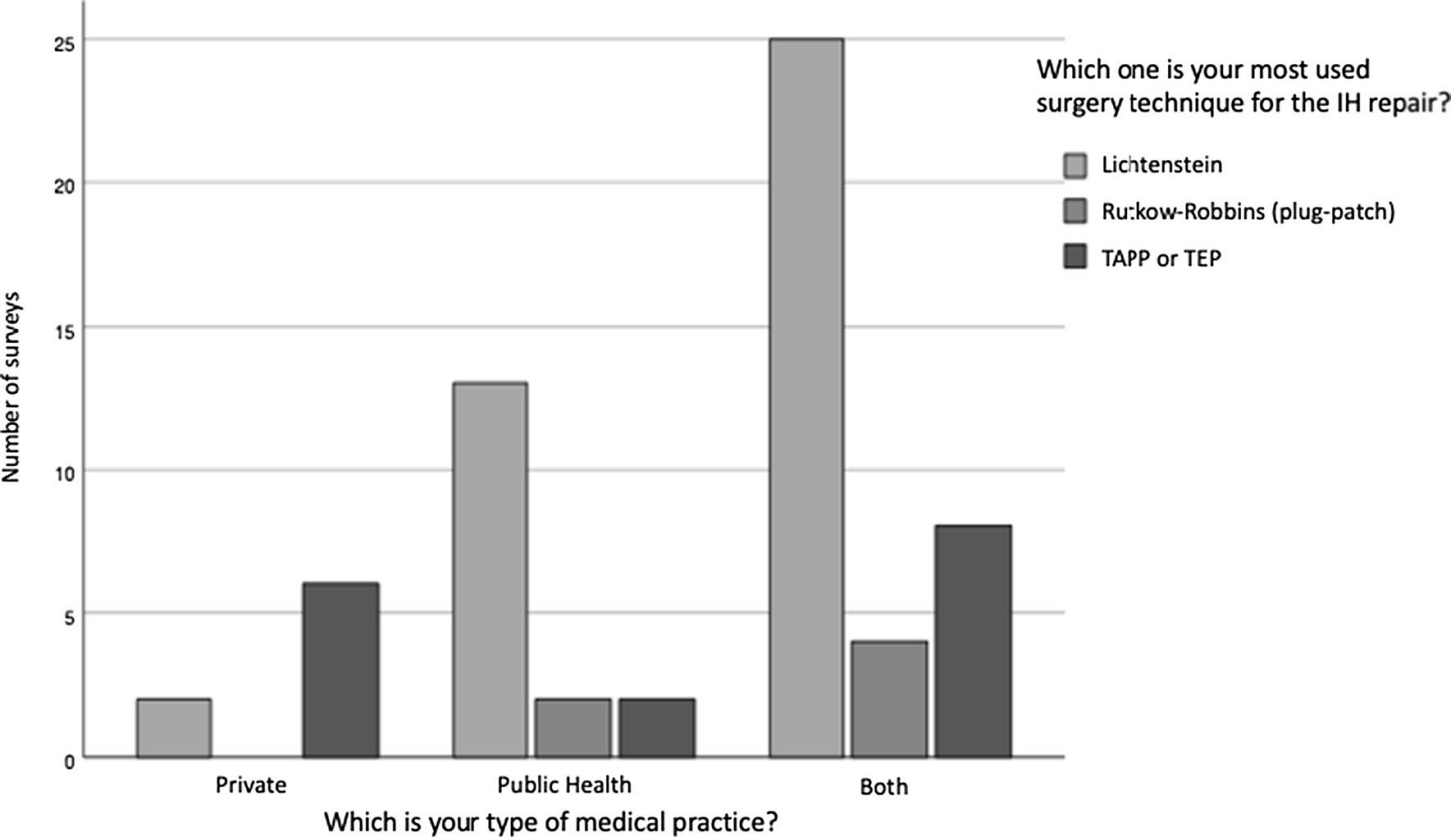
Association between “the type of medical practice” and “the most used surgery technique for IH repair”

There was a statistically significant association between the type of medical practice and the most used surgery technique for the IH repair (c^2^(fd 2) = 10.5, p < 0.05, Phi = 0.43, V Cramer = 0.43). On this question the Rutkow–Robbins technique had to be eliminated due to the expected amounts were very low. Figure 5 shows the frequencies.

The knowledge of the International Guideline was significantly related to the Progrip® use (c^2^(fd 2) = 14.3, p < 0.05, Phi = 0.48, V Cramer = 0.48). Remaining associations did not result in statistical significance or the test was invalidated by low expected counts.

## Discussion and comparison with 2018 International Guideline

A vast amount of information and investigation on inguinal hernia management is available on the internet, and it becomes difficult to recognize which have clinical relevance and impact. The development of clinical guidelines provides systemization and patterns of management, guaranteeing the best results [1, 6, 7]. A guideline is a set of standards, criteria and specifications neither prescriptive nor directive, to approach a given task [2], and must close the gap between research and practice [9]. The elaboration of guides must follow a rigorous scientific process and be an easily understood guide accompanied by implementation strategies to assure clinical benefit [10, 11]. The 2018 International Guide had the best updated evidence and the expert’s recommendations, but to us it had a low diffusion in CR.

The survey group corresponded to attending surgeons that mostly perform hernia surgery, in San Jose, in both private and public hospitals. The majority of whom have more than 5 years working as specialists and with experience in hernia pathology. 69% achieved more than 52 inguinal hernia surgeries per year, with an annual maximum of 300. Every province of the country was represented as well as young surgeons showing the wide range of IH management. A weak point was the low participation of residents despite being distributed among the postgrad program. The majority of IH were ambulatory surgery, following the guide’s recommendations [1] and by all general surgeons. Only 16% had an abdominal wall surgery team. This type of groups concentrates more experience, numbers, ability, resolution and better outcomes [4, 12]. Our surgeons showed to have a high-volume IH repair, described as more than 25 IH repair per year per surgeon, or more than 126 IH repair per year in one hospital. A high-volume surgeon has better results related to recurrence, complications and re operations [13, 14].

The type of medical practice was associated significantly to the surgical technique but not to the volume of IH repairs. Those with both practices, public and private, reached 6 IH surgeries per week most likely due to the increase in working hours. Those with exclusive private practice showed a lower volume. Working in the public health system as well in the private practice was associated with more Lichtenstein repairs and more laparoscopic procedures (p < 0.05), most likely due to higher surgical volumes. The guideline recommends both approaches for inguinal hernias [1].

The type of anesthesia used for IH was different than the recommendations. Local anesthesia in open repairs have the lowest complication rate [1] but the preference for this technique in CR was 0%. Spinal anesthesia was the most commonly used (74%) despite the higher incidence of myocardial infarctions and acute urine retention [1]. 67% of surgeons know how to perform a groin hernia repair with only local anesthesia but prefer not to use it perhaps because of a comfort reason that was not questioned in the poll. The majority use direct nerve blockage or at least local anesthesia in the fascia and subcutaneous tissue which follow the recommendations [1].

Minimally invasive surgery is recommended if the resource is available with proper training. Less than half of the cases were IHMIS in the majority of the hospitals, but there was a 12% minority that answered that more than half of the IH cases were laparoscopically. Indications for IHMIS were the same as the guideline’s recommendations, but the women as indication was poorly selected, and they have the greatest benefits for posterior approaches and IHMIS [15]. 95% of those surveyed use the same approach for men and women but the condition could have different behavior for both genders.

Unilateral primary hernia in men is a weak indication for laparoscopic repair. There is enough evidence of IHMIS benefit in this group of patients but there are socioeconomic and cultural issues that make open repair the choice [1]. In our country 92% of hospitals where the surveyed surgeons belong perform IHMIS. Even if the volume of cases is low and could be concluded that not enough number of cases are reached to warrant good results, it is important to mention that the difference between low and high-volume surgeons despite significant is very small. The standardization of the technique and continuous training could make the results of low volume surgeons similar to the high-volume ones [16]. It is evident that IHMIS is widely accepted in CR as 68% of the surveyed know how to perform it. Even among those who don’t practice IHMIS the statement that all surgeons who make groin hernia repairs should do it was chosen.

The IHMIS is questioned for having similar Lichtenstein repair results despite its higher cost although it has better post-surgery recovery and less chronic pain [1, 17–19]. This type of repair hasn’t been as well established as laparoscopic cholecystectomy. The 2018 HerniaSurge Guidelines recommend it in women, young, athletes, considerable pre-operative pain and recurrences of anterior approach repairs. In CR, IHMIS was traditionally reserved only for expert surgeons, but this paradigm seems to be changing.

It is important to mention the lack of studies for chronic pain incidence and the little use of the recommended EHS IH classification. The majority of the surveyed surgeons believe to have very few cases of chronic pain but lack studies to support that belief. Up to 12% of patients with groin hernia repair may present chronic pain of variable intensity and 1% of those will carry lifelong untreatable pain which makes the need for national studies urgent [20].

Regarding conservative management, no consensus with the recommendations was reached. In a patient that approves the conservative management it will be appropriate if the IH is small and minimally symptomatic or asymptomatic although 70% of the cases might end up in surgery within five years [1]. The surveyed agreed that if the diagnosis of the IH is made with sonography and without symptoms, they would not recommend surgery.

The International Guidelines recommend the surgical approaches with more support on the poll: Lichtenstein (64%) and TAPP and TEP laparoscopic (26%). The Rutkow–Robbins technique [21], the anatomic techniques and the posterior open approaches showed a low index of utilization. If the resource or the experience is not available for IHMIS, the Lichtenstein is an adequate technique for most of the cases although the ideal would be the tailored approach according to the type of hernia, the patient and the hospital [1, 22].

Management of the hernia sac was made according to the principles recommended: balance decision of resection of the sac and its reduction. A big sac reduced will recur more often and the systematic resection of the sac will show more postoperative pain (low level of evidence). The details not shared with the guidelines was the pubic tubercle fixation and cone mesh. Also, with low evidence is recommended the non-fixation of the mesh to the periosteum of the pubic tubercle because it could induce chronic pain. The cone mesh should be avoided given they don’t diminish recurrences and could migrate or erode close organs and also, they produce fibrosis in multiple layers of the inguinal canal [1].

In the latest meta-analysis, there are no differences between groin prosthesis fixation mechanisms. In theory the non-absorbable sutures can cause chronic pain, so experts recommend slow absorbing sutures such as polydioxanone or polyhydroxybutyrate. It is now recommended to fix avoiding tissue trauma to diminish the incidence of postoperative pain [1]. The guidelines state Progrip® mesh has no advantage related to pain although more studies are needed on this area. The Costa Rican surgeons use this latest mesh and recognize its advantages and disadvantages as well. The guide recommends the use of synthetic glues to lower the acute and chronic pain incidence and despite the fact that these devices are not generalized, 65% of the surgeons are willing to use it in the IH [1].

The pre peritoneal open hernia repair [23] was the great unknown of the poll and despite 24% of the surveyed indicated they use it as the standard or in IH anterior recurrences this statement was not checked in other questions of the same topic. Repairs on this space have wide variety and few well-designed studies which is why they are not recommended but its huge potential is recognized. The IHMIS offers better visibility and security but comparing the results with open pre peritoneal repairs there are big similarities [1]. In areas of low resources as well as for femoral hernias this technique could be a good substitute for IHMIS.

The emergency hernia management was similar to the recommendations. The use of mesh in the strangulated IH should be done according to the level of contamination and the best approach is the one that resolves the emergency although there is no optimal evidence [1].

The interest in the update of IH management was high. Most of the surveyed surgeons have updated courses and 92% wish to improve the surgical technique. And still 52% of the surgeons had no knowledge of the 2018 international guidelines despite being a trained and experienced group in IH surgery indicating low diffusion in the country. From the 46% who knew the existence of the guidelines only 18% use it as a reference indicating poor use. The guidelines should be an essential technical background for all IH surgeons. Educational activities should be organized to ensure the diffusion of the information and place ourselves among the international standards [24] however implementation might be slow if academic efforts are not carried out to change the clinic practice [6, 25, 26]. Knowledge of the guidelines was only related to the use of Progrip® mesh and did not relate to the other questions. The number of surveys was an important limitation, maybe with a higher volume, other relations could have been found.

An expert in hernia disease is the surgeon who has sufficient skills and volume of cases to train and educate and who investigates within the field [1, 4]. Considering oneself an expert was only associated to the technique used but not to the volume of IH surgeries. The self-proclaimed experts perform more IHMIS and the non-experts perform more Lichtenstein (Fig. 4). The number of participants influenced and could be explained that to consider oneself an expert for the Costa Rican surgeons relates to the individual preparation and not to a high volume of cases.

Despite little knowledge of the 2018 Guidelines, the participating surgeons follow them partially. These Guidelines are not legal regulations and are adjustable depending on the resources, abilities and preferences of the surgeon as well as the patient. There should be educational activities and results assessment and further investigation to improve training [1, 27]. The most likely bias of this work could be the survey is based on opinion and perception. Most of the participants don’t have data to support their selections. Ideally the inquiry should have been taken by all general surgeons performing groin hernias. Despite the efforts and diffusion of the inquiry in the country the most representative group was from the country capital. There can also exist a bias of interpretation because some questions were formulated towards the hospital setting and not directly to the surgeon.

## Conclusions

Despite little knowledge of the 2018 International Guidelines, the surveyed surgeons followed partially its recommendations. The majority of the surgeons comply in relations to outpatient procedure and emergency surgery, but the local anesthetic technique is not used except to support the postoperative analgesia. There is a wide acceptance and knowledge of laparoscopic-endoscopic groin hernia management but in the open procedure, which is the most widely performed, not all the recommendations are followed. The pre peritoneal open hernia repair was the least known technique. Considering hernia experts should be able to perform the best tailored approach to every patient according to the defect size and co morbitities, there should be hands on courses emphasizing this particular technique. It must be a priority for real development to be monitored and evaluated along with the validation of results and further investigation.

The International Guideline for the Groin Hernia Management from 2018 shows little diffusion among surgeons in CR so more academic activities should be encouraged for their proper application. The overwhelming desire to improve is the motivation for future academic activities. Local protocols according to the national situation and based on the 2018 Guidelines should be formulated as recommended by the Hernia Surge Group.

## Supplementary Information

**Additional file 1. **Survey inguinal hernia management in Costa Rica.

## Data Availability

The datasets used and/or analyzed during the current study are available from the corresponding author on reasonable request.

## Abbreviations

IH: Inguinal Hernia
EHS: European Hernia Society
AMH: Mexican Hernia Association
IHMIS: Inguinal hernia minimally invasive surgery
TAPP: Pre peritoneal repair
TEP: Total extra peritoneal repair
CR: Costa Rica
