# Supplementary material for: The inguinal hernia management in Costa Rica according to a survey between surgeons: result comparison with 2018 International Groin Hernia Guidelines
Source: BMC Surg. 2021 Mar 21;21:152. doi: 10.1186/s12893-021-01156-9 (PMC7981829; doi:10.1186/s12893-021-01156-9)
Supplement: Supplementary file 1 — Additional file 1. Survey inguinal hernia management in Costa Rica. [file 12893_2021_1156_MOESM1_ESM.docx]

ANNEXUM

SURVEY "INGUINAL HERNIA MANAGEMENT IN COSTA RICA"

1. Are you a general surgeon?

a. Yes

b. No

c. Resident

2. Where do you practice surgery?

a. San José

b. Alajuela

c. Puntarenas

d. Heredia

e. Limón

f. Guanacaste

g. Cartago

3. Which is your type of medical practice?

a. Private

b. Public

c. Both

4. How many years have you been practicing as a specialist?

a. Less than 5 years

b. Between 5 and 10 years

c. More than 11 years

5. Which is your most common surgery?

a. Colorectal

b. Esophagogastric

c. Endocrine and metabolic

d. Hepatobiliopancreatic

e. Abdominal wall surgery

F. General Emergency Surgery

g. Other

6. How many inguinal hernia surgeries do you do in a week?

a. Less than 1

b. 1 to 3

c. 4 to 5

d. More than 6

7. In your hospital, who do the inguinal hernia (IH) repairs?

a. A team / group

b. All the surgeons

c. The resident mostly

8. In your hospital, how many IH are operated on an ambulatory setting?

a. Few

b. Less than half

c. The majority

9. In your hospital, IH is generally performed under anesthesia...

a. General

b. Spinal anesthesia

c. Local and sedation

10. Do you use a inguinal hernia classification?

a. No

b. Direct or Indirect

c. Gilbert

d. Nyhus

e. EHS

f. Other

11. To a young male patient with asymptomatic or minimally symptomatic small inguinal hernia, would you offer a management with observation and periodic appointments?

a. Yes

b. No

12. Are you comfortable performing a surgery in a young male patient with an asymptomatic nonpalpable hernia diagnosed by ultrasound?

a. Yes

b. No

13. What is your most used technique for the IH treatment?

a. Lichenstein

b. Rutkow Robbins (plug and patch, plugstein, cone and mesh)

c. Nyhus or open preperitoneal techniques

d. Shouldice or anatomical techniques.

a. TAPP or TEP

14. Do you use a mesh cone on indirect hernias?

a. Yes

b. No

c. Sometimes

15. Do you accomplish local anesthetic infiltration for inguinal hernioplasties in a patient with

general or spinal anesthesia?

a. Yes

b. No

16. Regarding IH surgery with local anesthesia and sedation, at your hospital

a. It is used only in small hernias and young patient

b. It is used in older adults, with significant comorbidities

c. We do not normally use this anesthetic option

17. In your hospital the direct nerve block in IH surgery

a. We don't do blockage, maybe skin infiltration and TCS

b. The anesthetist does it before or after the intervention

c. The surgeon does it during the intervention

18. Do you know how to perform an open inguinal hernioplasty with only local anesthesia?

a. Yes

b. No

19. In a woman with an IH, would you give "observation" without performing surgery?

a. Yes

b. No

20. In a woman with IH, do you use the same technique and approach as in a man?

a. Yes

b. No

21. For an indirect hernia sac, what would you do?

a. I never resect it out

b. I always resect it out

c. I only resect it if its big

22. How often do you do laparoscopic inguinal hernia surgery?

ta. I do not do laparoscopic inguinal hernioplasty

b. Less than 1 case a week

c. 1 case per week

d. 2 to 3 cases a week

e. More than 3 cases per week

23. In your hospital, how many IH are operated laparoscopically?

to. We don't do IH laparoscopy

b. Very few

c. Less than half of the cases

d. More than half of the cases

24. In your hospital, which are the indications for laparoscopic inguinal hernioplasty? (In this question you can select several answers)

a. Bilateral IH

b. IH recurred

c. HI in women

d. HI in athletic patients

e. Primary unilateral IH in men

25. How do you secure the meshes in an IH open surgery?

a. With cyanoacrylate

b. With self-adhesive meshes (Progrip)

c. With Nylon suture

d. With Polypropylene suture

e. With short-term absorbable suture, like vicryl suture

f. With long-term absorbable suture, like PDX suture

g. I don't secure the mesh

26. In a Lichtenstein-type inguinal hernia mesh repair, do you hold the mesh to the pubic tubercle periosteum?

a. Yes

b. No

27. Do you currently use self-adhesive meshes like Progrip in the IH repair?

a. Yes

b. No

28. Your opinion about the ADVANTAGES of the Progrip mesh (In this question you can select SEVERAL ANSWERS)

a. Less chronic pain

b. Less operative time

c. Easy handling

d. Can be trimmed to fit size

e. It offers no advantages

Your opinion about the DISADVANTAGES of the Progrip mesh (you can select MULTIPLE ANSWERS)

a. Higher cost

b. Increased foreign body sensation

c. It does not have an adequate cost / benefit ratio

d. Difficult handling at the surgical act

e. It has no disadvantages

29. Do you use, or would you use a synthetic / biological glue as a substitute for mesh anchor points in an open inguinal hernioplasty in a patient with a small hernia?

a. Yes

b. No

30. In your hospital, are there chronic pain cases after IH surgery?

a. Yes, there are a few cases, but we do not have incidence studies

b. There are no cases, although we do not have incidence studies

c. We have studies and it is below 10%

d. We have studies and it is below 15%

31. In relation to open preperitoneal hernioplasty

a. I have no experience in this approach

b. I use it in cases of anterior hernioplasty recurrence

c. I only do it in the emergency room

d. I always use it as standard

32. Your approach to incarcerated IH in the emergency room

a. I do conventional open surgery

b. I do laparoscopic approach

c. I prefer the open preperitoneal approach

33. If an incarcerated IH is reduced in the emergency room, the next step in your hospital is:

a. Deferred urgent surgery (2-3 days later we perform the surgery)

b. Refer to priority waiting lists. (short waiting time)

c. Refer to ordinary waiting lists. (long waiting time)

34. In the case of a strangled IH, what do you do?

a. Avoid mesh because of the infection risk

b. Depending on the contamination, I place a mesh

c. Always use mesh

d. I do not operate emergency cases.

35. If you are going to do a unilateral open inguinal hernioplasty in a healthy patient, would you indicate prophylactic antibiotics?

a. Yes

b. No

36. In an elective patient with a femoral hernia, your preferred approach is:

a. McVay

b. Lichtenstein with femoral modification

c. TAPP or TEP

d. Mesh cone in femoral orifice

e. Open posterior approach like Nyhus

37. Do you know the 2018 International Clinical Guide for the Inguinal Hernia management from Hernia Surge Group?

a. I have not heard of it

b. I have read some of it,

c. I have read the guide and I don't like it

d. I have read it and it serves as a reference

38. Do you want to improve your surgical technique in inguinal hernioplasties?

a. Yes

b. No

39. Do you think laparoscopic inguinal hernia surgery should be done by all surgeons who operate inguinal hernias?

a. Yes

b. No

40. Do you consider yourself as an expert in the management and treatment of abdominal wall hernia surgery?

a. Yes

b. No

41. Do you consider yourself as an expert in the management and treatment of hernia surgery of the inguinal hernia?

a. Yes

b. No

42. Your opinion about your training and updating in HI surgery:

a. I learned well as a resident and I don't need to learn more

b. I would like to be a little more updated

c. I have taken courses to improve and be updated

d. I am not interested in this pathology
